# Dissecting *TSC2*-mutated renal and hepatic angiomyolipomas in an individual with *ARID1B*-associated intellectual disability

**DOI:** 10.1186/s12885-019-5633-1

**Published:** 2019-05-10

**Authors:** Bernt Popp, Abbas Agaimy, Cornelia Kraus, Karl X. Knaup, Arif B. Ekici, Steffen Uebe, André Reis, Michael Wiesener, Christiane Zweier

**Affiliations:** 1 Institute of Human Genetics, Friedrich-Alexander-Universität Erlangen-Nürnberg (FAU), Schwabachanlage 10, 91054 Erlangen, Germany; 2 Institute of Pathology, University Hospital Erlangen, Friedrich-Alexander-Universität Erlangen-Nürnberg (FAU), 91054 Erlangen, Germany; 3 Department of Nephrology and Hypertension, Friedrich-Alexander-Universität Erlangen-Nürnberg (FAU), 91054 Erlangen, Germany

**Keywords:** ARID1B, TSC2, Tuberous sclerosis complex, Neurodevelopmental disorders, Angiomyolipoma, Mosaic

## Abstract

**Background:**

Several subunits of the SWI/SNF chromatin remodeling complex are implicated in both cancer and neurodevelopmental disorders (NDD). Though there is no clinical evidence for an increased tumor risk in individuals with NDDs due to germline mutations in most of these genes so far, this has been repeatedly proposed and discussed. A young woman with NDD due to a de novo mutation in *ARID1B* now presented with a large renal (> 19 cm in diameter) and multiple hepatic angiomyolipomas (AMLs) but no other signs of tuberous sclerosis complex.

**Methods:**

We analyzed tumor and healthy tissue samples with exome and panel sequencing.

**Results:**

Additionally to the previously known, germline *ARID1B* variant we identified a post-zygotic truncating *TSC2* variant in both renal and hepatic AMLs but not in any of the healthy tissues. We did not detect any further, obvious tumor driver events. The identification of a passenger variant in *SIPA1L3* in both AMLs points to a common clonal origin. Metastasis of the renal AML into the liver is unlikely on the basis of discordant histopathological features. Our findings therefore point to very low-grade mosaicism for the *TSC2* variant, possibly in a yet unknown mesenchymal precursor cell that expanded clonally during tumor development. A possible contribution of the germline *ARID1B* variant to the tumorigenesis remains unclear but cannot be excluded given the absence of any other evident tumor drivers in the AMLs.

**Conclusion:**

This unique case highlights the blurred line between tumor genetics and post-zygotic events that can complicate exact molecular diagnoses in patients with rare manifestations. It also demonstrates the relevance of multiple disorders in a single individual, the challenges of detecting low-grade mosaicisms, and the importance of proper diagnosis for treatment and surveillance.

**Electronic supplementary material:**

The online version of this article (10.1186/s12885-019-5633-1) contains supplementary material, which is available to authorized users.

## Background

*ARID1B* haploinsufficiency (OMIM #135900, *614556) represents a frequent cause for neurodevelopmental disorders (NDD) [1] comprising nonspecific intellectual disability [2] or Coffin-Siris syndrome [3, 4]. The AT-rich interactive domain-containing protein 1B encoded by the *ARID1B* gene is a subunit of the SWI/SNF (SWItch/Sucrose Non-Fermentable) chromatin remodeling complex. This complex and many of its components play an important role in a broad spectrum of neoplasms, acting mainly as tumor suppressors [5]. *ARID1B* therefore belongs to a growing number of genes in which germline mutations cause NDDs while somatic mutations are involved in cancer [6]. Though there is no clinical evidence that individuals with NDDs due to germline mutations in most of these genes and particularly in *ARID1B* bear an increased risk for tumors [6, 7], this has been repeatedly proposed and discussed [3, 8].

We report on a young woman previously diagnosed with intellectual disability due to a de novo mutation in *ARID1B*, in whom a huge renal and multiple hepatic angiomyolipomas (AMLs) were diagnosed. Investigating their molecular cause revealed a post-zygotic stop-variant in *TSC2* in addition to the previously known germline variant in *ARID1B*.

## Methods

### Clinical report

**Fig. 1:**
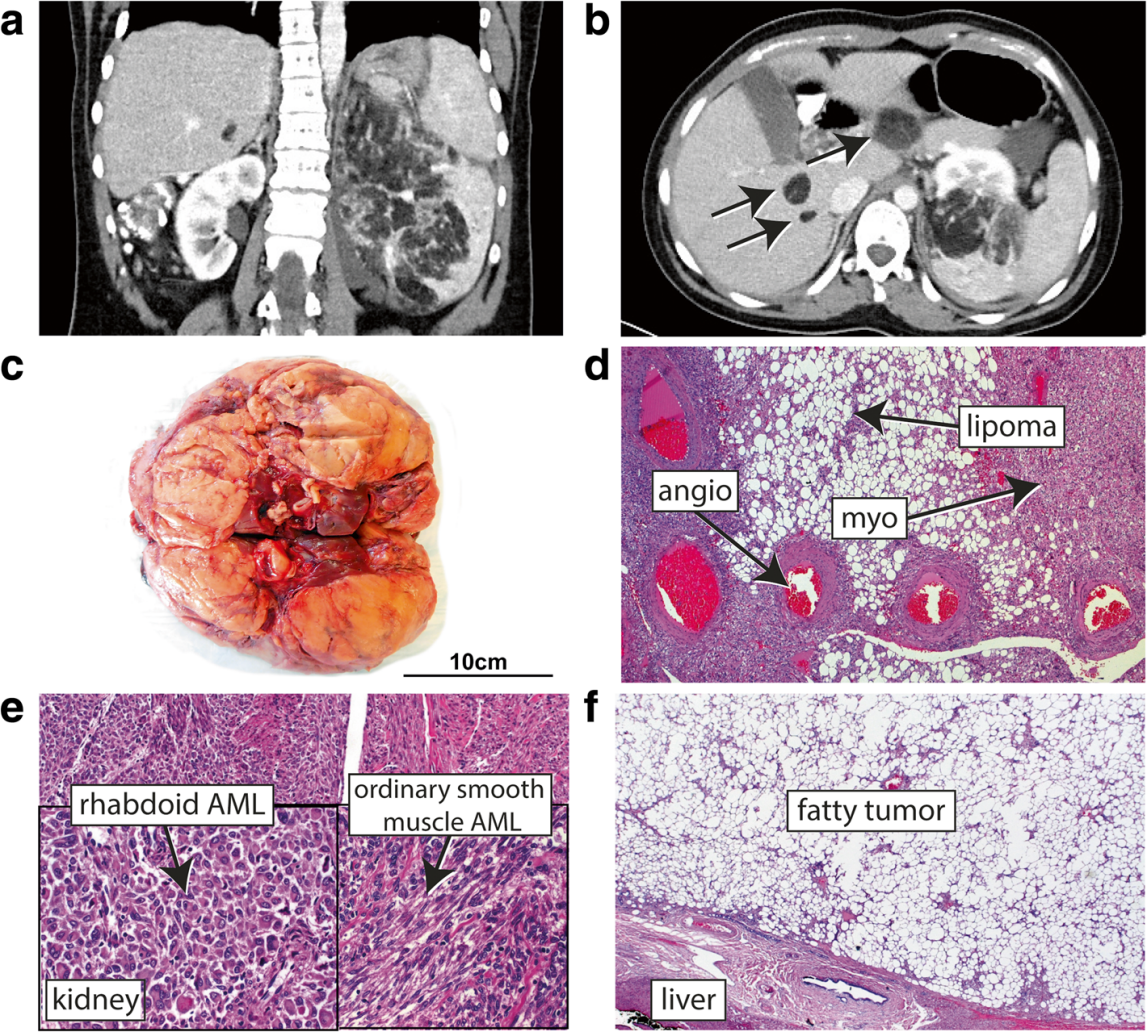
Renal and hepatic AMLs. **a** Abdominal CT scan showing the large AML of the left kidney. **b** CT of the liver showing three lesions indicated by arrows. **c** Macroscopic picture of the renal AML. **d** Histopathology with haematoxylin and eosin (HE) staining showing blood vessels lacking elastic tissue (“angio”), smooth muscle cells (“myo”) and adipose tissue (“lipoma”) close together as a typical sign of angiomyolipoma. **e** HE staining of a region from the large kidney AML showing the ordinary smooth muscle differentiation and rhabdoid/epithelioid cell features. **f** HE staining of a region from the resected hepatic AML, being composed mainly of fat cells

This individual had been reported previously (patient 5) when the de novo variant c.3304C > T, p.(Arg1102*) in *ARID1B* (NM_020732.3) was identified to be causative for her intellectual disability [2]. Clinical details up to 12 years 8 months are described elsewhere [2]. At age 19 she had moderate cognitive impairment with good comprehension, speaking in two-word sentences and friendly behavior. After indicating abdominal pain, examination by ultrasound and computer tomography (CT) revealed a large (diameter 19 cm) tumor in the left kidney and multiple liver tumors (> 8, average diameter ca. 2 cm) (Fig. 1a-c). Histological investigation after nephrectomy and removal of one of the liver tumors confirmed them to be AMLs (Fig. 1d-f). Subsequent cranial MRI (magnetic resonance imaging), ultrasound of the right kidney and detailed skin and nail examination did not reveal any further clinical features of tuberous sclerosis complex such as subependymal nodules or cortical dysplasia, skin lesions or ungual fibromas. Apart from two single seizures in early infancy she had no history of epilepsy. Meanwhile, cardiologic evaluation and computer tomography of the lung did not reveal further anomalies.

### DNA extraction and sequencing

DNA from peripheral blood lymphocytes (PBL) was extracted by standard procedures. DNA from cultured fibroblasts (obtained from a cleft palate surgery specimen) and from renal tubular cells grown from a urine specimen of the remaining healthy kidney as described by others [9] were extracted using the Qiagen DNAeasy system according to manufacturer’s recommendations (Qiagen, Hilden, Germany).

Genomic DNA from marked tumor and neighboring non-neoplastic tissues was extracted from 5 μm sections. After de-paraffinization the NucleoSpin Tissue kit was used according to the manufacturer’s protocol (Macherey-Nagel, Düren, Germany), and subsequently lysis with proteinase K at a concentration of 0.8–1.0 U per digest at 55 °C overnight was performed. Exome sequencing of DNA from blood, renal and liver tumor was performed on an Illumina HiSeq 2500 system (Illumina, Inc., San Diego, USA) after enrichment with the SureSelect Target Enrichment v6 technology (Agilent Technologies, Santa Clara, CA). Sequencing of DNA from unaffected renal and hepatic tissues and from fibroblasts and renal tubular cells was performed on an Illumina MiSeq system using the TruSight Cancer panel. Complete coverage (>20x) for *TSC1* (NM_000368.3) and *TSC2* (NM_000548.3) was ensured. MLPA analysis for *TSC1* and *TSC2* on PBL DNA was performed using the kits P046 and P124 from MRC-Holland (Amsterdam, The Netherlands). Variants in *TSC2* and *SIPA1L3* (NM_015073.2) were validated by Sanger sequencing using standard procedures.

Resulting sequencing reads were aligned and processed as described previously [10]. Concurrent variant calling of the two AML and the PBL exome BAM files together with 13 control samples from the same machine run was performed using freebayes version 1.1.0 [11]. SnpEff/SnpSift version 4.3p [12] with dbNSFP [13] was used for annotation. Potential somatic variants at coding and splice region positions from the freebayes calls were filtered to have an alternative allele fraction (AF) ≤1% in blood and of ≥5% in the respective tumor. Only sites with a read coverage of ≥10 in each sample and with ≥5 alternative allele reads in the tumor were considered. Additionally, variants present with an allele depth of ≥2x in 13 in-house controls from the same run were excluded. Copy number variation (CNV) screening from exome data was done with CNVkit version 0.9.4.dev0 [14]. BAM files were then visually inspected at regions of interest using the IGV browser version 2.4.7 [15, 16].

## Results

**Fig. 2:**
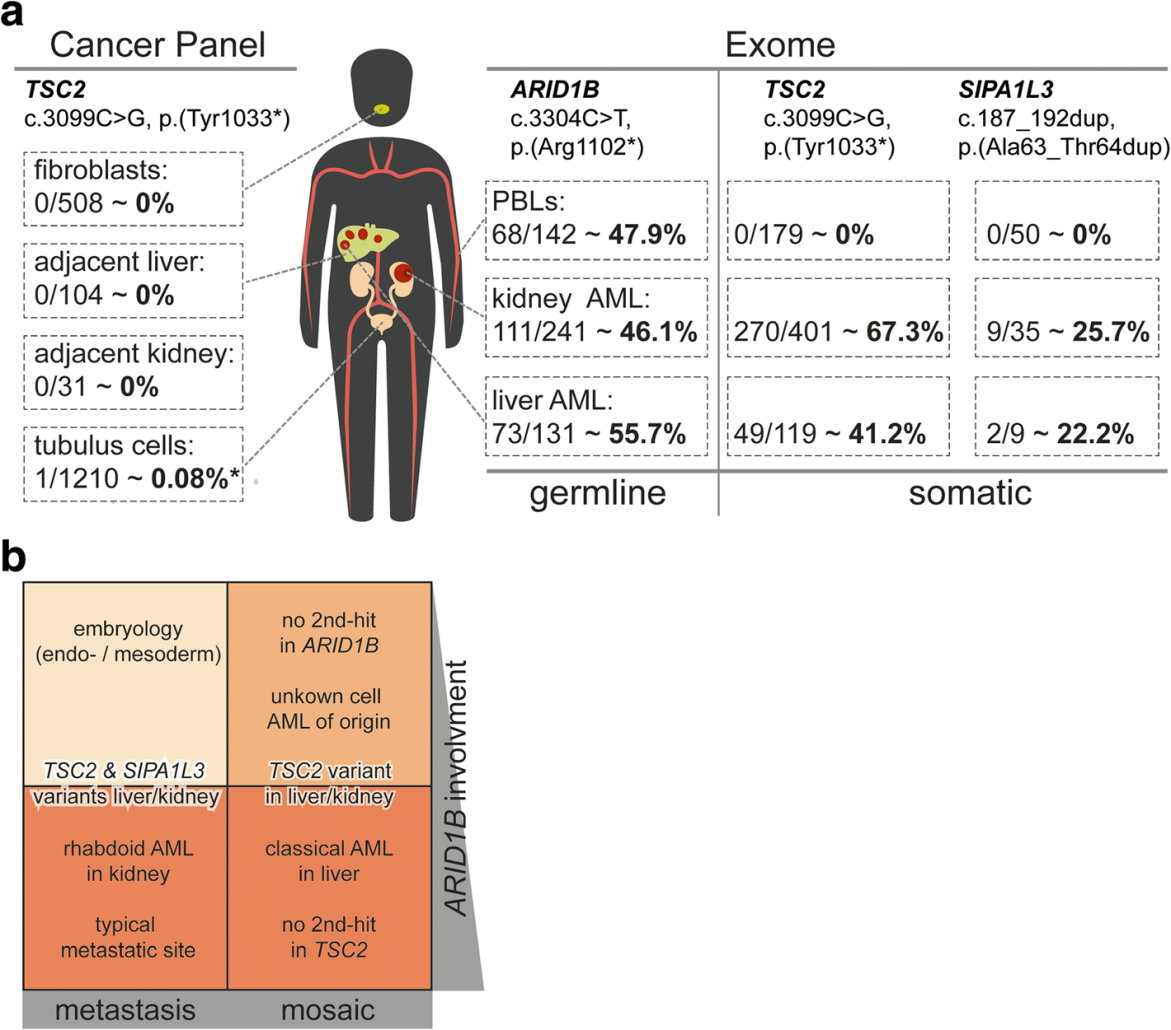
Molecular analyses on various tissues. **a** Overview of the analyzed tissue samples using exome, panel or Sanger sequencing and the results for the respective genomic regions of interest. Both somatic variants in *TSC2* and *SIPA1LR* were identified and confirmed. No evidence for complete LOH at the *ARID1B* and *TSC2* loci was found in the AML samples. Also see Additional file 1: Figures S1, S4 and S5 for details including IGV snapshots and Sangers sequencing results. **b** Schematic 2 × 2 contingency table of the different possible hypotheses for the tumor formation with arguments supporting each of the four combinations; the color intensity encodes perceived likeliness. VAF, variant allele frequency; CR, copy ratio; *: this one read is likely an artefact, compare Additional file 1: Figure S5

Analysing exome data from blood and renal and hepatic AMLs showed the known variant in *ARID1B,* as expected, in about 50% of the reads, respectively (Fig. 2a and Additional file 1: Figure S1). No further pathogenic variant in *ARID1B,* nor loss of heterozygosity (LOH) was observed in tumor tissues (Additional file 1: Figures S2 and S3). Analysis of *TSC1* and *TSC2* revealed the pathogenic variant c.3099C > G, p.(Tyr1033*) [17] in *TSC2* (NM_000548.4) in 67.3% (270/401) of the reads in DNA from the renal and in 41.2% (49/119) of reads from the hepatic AML, while it was not detected in any of the 179 reads from blood (Fig. 2a and Additional file 1: Figure S1). Panel sequencing on DNA from unaffected renal and liver tissues and from fibroblasts did not detect the *TSC2* variant in any of the 31 to 508 reads, respectively (Fig. 2a and Additional file 1: Figure S4), but in one of 1210 reads in DNA from tubular cells. However, given its location at the beginning of the read and as it occurred in only one read in an overlapping read pair, it most likely represents an artefact (Fig. 2a and Additional file 1: Figure S5).

Searching exome data for additional variants in renal or hepatic AML and absent in blood revealed a variant in *SIPA1L3* (c.187_192dup, p.(Ala63_Thr64dup) (NM_015073.2); COSM5959440), which was additionally excluded by Sanger sequencing in DNA from fibroblasts and healthy renal and hepatic tissues. Dysfunction of SIPA1L3 has been discussed as a possible contributor to the phenotype of some malignancies as it led to abnormalities of epithelial cell morphogenesis, cytoskeletal structure and adhesion in a colorectal adenocarcinoma cell line and as somatic *SIPA1L3* alterations were found in various, mainly epithelial cancer types [18, 19]. However, it has not yet been reported as a tumor driver ( [20] and Cosmic Cancer Gene Census (CGC)), and it was not found to be mutated in a study searching for additional somatic mutations in TSC-related lesions [21]. Therefore, we consider this variant most likely a passenger variant, though we cannot exclude a contributing effect in the tumor pathogenesis in our patient. Copy number analysis from exome data indicated a possible deletion of *TSC2* in a subpopulation of tumor cells. However, analysis of heterozygous variant positions at this locus did not show a significant deviation from 0.5, therefore not supporting validity of this finding (Additional file 1: Figures S2 and S3 and Additional file 2). These analyses are challenged by variability in exome data (e.g. coverage uniformity) and compromised tumor purity. No other large regions of LOH or CNVs were detected in the kidney or liver AMLs, indicating a relatively stable constellation in both tumors (Additional file 1: Figures S2 and S3; Additional file 2).

## Discussion

Tuberous Sclerosis Complex (TSC; OMIM #191100, *605284, #613254, *191092) is a multisystemic disorder generally caused by germline variants in *TSC1* or *TSC2*. Multiple renal and extra-renal angiomyolipomas (AMLs) represent one of the characteristic manifestations of TSC. Renal AMLs are commonly observed in 80% of patients, while hepatic AMLs are less frequent and occur in ca. 13% of affected individuals [22]. When renal and hepatic AMLs were detected in the herewith reported individual with *ARID1B* associated NDD and without other frequent and typical signs of TSC, we considered various possibilities: a) The AMLs might be causally related to the germline *ARID1B* mutation. b) The AMLs represent a mild and atypical manifestation of TSC in the sentence of multiple diagnoses as reported recently in a large study that revealed aberrations in two or more disease loci in 4.9% of 7374 individuals with various phenotypes tested by clinical exome sequencing [23]. Or c) Another cause, un-related to the known *ARID1B* variant and not necessarily deducible from the AML phenotype might be responsible for the unusual presentation.

When performing exome sequencing on DNA from the renal and hepatic AMLs, we found no indication for a contribution of *ARID1B* or other commonly cancer-related genes such as *TP53* to their genesis. While exome sequencing on blood did not detect a variant in the TSC genes *TSC1* or *TSC2*, we detected the same pathogenic *TSC2* variant in both renal and hepatic tumor tissues. This and an additional variant in *SIPA1L3* also exclusively found in both AMLs underline their common clonal origin and open two possibilities: a) the multiple hepatic AMLs being metastases from the huge renal AML as rarely reported particularly for epithelioid AMLs [24], or b) the AMLs resulting from a low-grade mosaicism for the *TSC2* variant. The first possibility seems extremely unlikely based on the discordant histopathological features of the AMLs. The huge renal tumor was predominantly composed of epithelioid cells entrapping only a very minor fatty component. In contrast, the resected hepatic AML was almost devoid of such epithelioid cells and was composed almost exclusively of fat cells closely mimicking a lipoma. Further supporting the second possibility, mosaicism has been frequently observed for *TSC2* variants, including low-grade mosaicism with allele frequencies < 1% or variants only detected in skin tumor biopsies but not in blood or saliva [25].

Of note, we did not reliably observe the *TSC2* variant in any of the sequencing reads from unaffected kidney, unaffected liver, fibroblasts or renal tubular cells, which might be expected in case of mosaicism. Given that the lesional cell of these *TSC*-related mixed neoplasms, the so-called “perivascular epithelioid cell” (PEC) has no known physiological counterpart, renal and hepatic AMLs might have their origin in a common, yet unknown mesenchymal precursor cell that expanded clonally during tumor development [26]. This has previously been discussed for the co-occurrence of sporadic pulmonary lymphangioleiomatosis and renal AMLs with somatic *TSC2* variants [27] and for TSC-associated AMLs [28]. Presence of the *TSC2* variant in only very few tumor precursor cells might therefore hinder detection in healthy tissues even by next-generation-sequencing (overview in Fig. 2b).

In contrast to many other tumors, AMLs are genetically relatively stable, and bi-allelic loss of *TSC2* has been postulated as a sufficient and the main driver for AML development [28]. Given the absence of a second *TSC2* variant, *TSC2*-LOH or any other evident tumor driver apart from the unclear variant in *SIPA1L3* in the renal and hepatic AMLs of the herewith reported individual, a contribution of the germline *ARID1B* variant to the tumorigenesis cannot be excluded.

In any case, identifying the *TSC2* variant in the herewith reported individual has important consequences for her medical care. It now prompts TSC specific surveillance including regular echocardiograms, high-resolution computed tomography of the lung and brain MRIs in addition to regularly checks of the remaining kidney and the liver. As mTOR inhibitors have been proven to be an efficient therapy in TSC [29], this treatment option will be evaluated and discussed based on her future disease course and manifestations.

Despite a good sequencing depth of 179 reads the *TSC2* variant was not detectable in blood, meaning that it also would not have been detected as an incidental finding when performing exome sequencing because of the NDD and thus would not have resulted in predictive counseling and surveillance procedures. Proper medical care in individuals with mental handicap is often challenging due to limited expression of medical discomfort and thus missed co-morbidities or health complications unrelated to the initial diagnosis.

## Conclusion

Next to the value of combining genetic and pathologic findings, this unique case demonstrates and emphasizes several generally important aspects of genetic and medical care such as a) considering multiple (independent) diagnoses in a single individual, b) considering mosaicism or post-zygotic variants and taking the efforts to detect them, as c) a proper diagnosis might have important consequences on treatment and surveillance. These efforts can be challenged by potential mosaicism in multi-tumor syndromes where the mutation-carrying cell of origin is still unknown and might represent a very minor yet unidentifiable cell population leading to false-negative results.

## Additional files

Additional file 1: **Figure S1**. IGV snapshots from exome sequencing. **Figure S2**. Copy number analysis from exome data from the kidney AML using CNVkit. **Figure S3**. Copy number analysis from exome data from the liver AML using CNVkit. **Figure S4**. IGV snapshots from targeted Cancer Panel and Sanger sequencing. **Figure S5**. IGV snapshot of the artefact read identified in tubulus cells. (DOCX 1912 kb)

Additional file 2: Exome CNV calls and heterozygous SNPs in the *ARID1B* or *TSC2* region. Microsoft Excel spreadsheet file containing the worksheets “summary”, “CNVkit_exome-aberrations” and “LOH_ARID1BandTSC2”. The “summary” worksheet contains a detailed description of all other worksheets and the respective data columns. The “CNVkit_exome-aberrations” worksheet contains all the segments called by CNVkit 0.9.4.dev0 which have a copy numer different from 2. The “LOH_ARID1BandTSC2” worksheet contains all heterozygous single nucleotide variants called in the PBL and both kindey and liver AML samples with at least 20 bp coverage in each sample (to reduce sampling bias) with 500 k bases of the *ARID1B* (chr6[hg19]:156599064–158,031,913) or *TSC2* (chr16[hg19]:1597990–2,638,713) gene. Fisher’s exact test from R version 3.4.3 was used to calculate *p*-values between the PBL and AML read coverages at each SNV position to check for significant deviation from the expected allele fraction. (XLSX 48 kb)

## Abbreviations

AML: Angiomyolipoma
CNV: Copy number variant
LOH: Loss of heterozygosity
MRI: Magnetic resonance imaging
NDD: Neurodevelopmental disorder
PBL: Peripheral blood lymphocytes
PEC: Perivascular epithelioid cell
SWI/SNF: SWItch/Sucrose Non-Fermentable
TSC: Tuberous sclerosis complex
