# Supplementary material for: Dissecting TSC2-mutated renal and hepatic angiomyolipomas in an individual with ARID1B-associated intellectual disability
Source: BMC Cancer. 2019 May 10;19:435. doi: 10.1186/s12885-019-5633-1 (PMC6511147; doi:10.1186/s12885-019-5633-1)
Supplement: Supplementary file 1 — Figure S1. IGV snapshots from exome sequencing. Figure S2. Copy number analysis from exome data from the kidney AML using CNVkit. Figure S3. Copy number analysis from exome data from the liver AML using CNVkit. Figure S4. IGV snapshots from targeted Cancer Panel and Sanger sequencing. Figure S5. IGV snapshot of the artefact read identified in tubulus cells. (DOCX 1912 kb) [file 12885_2019_5633_MOESM1_ESM.docx]

**
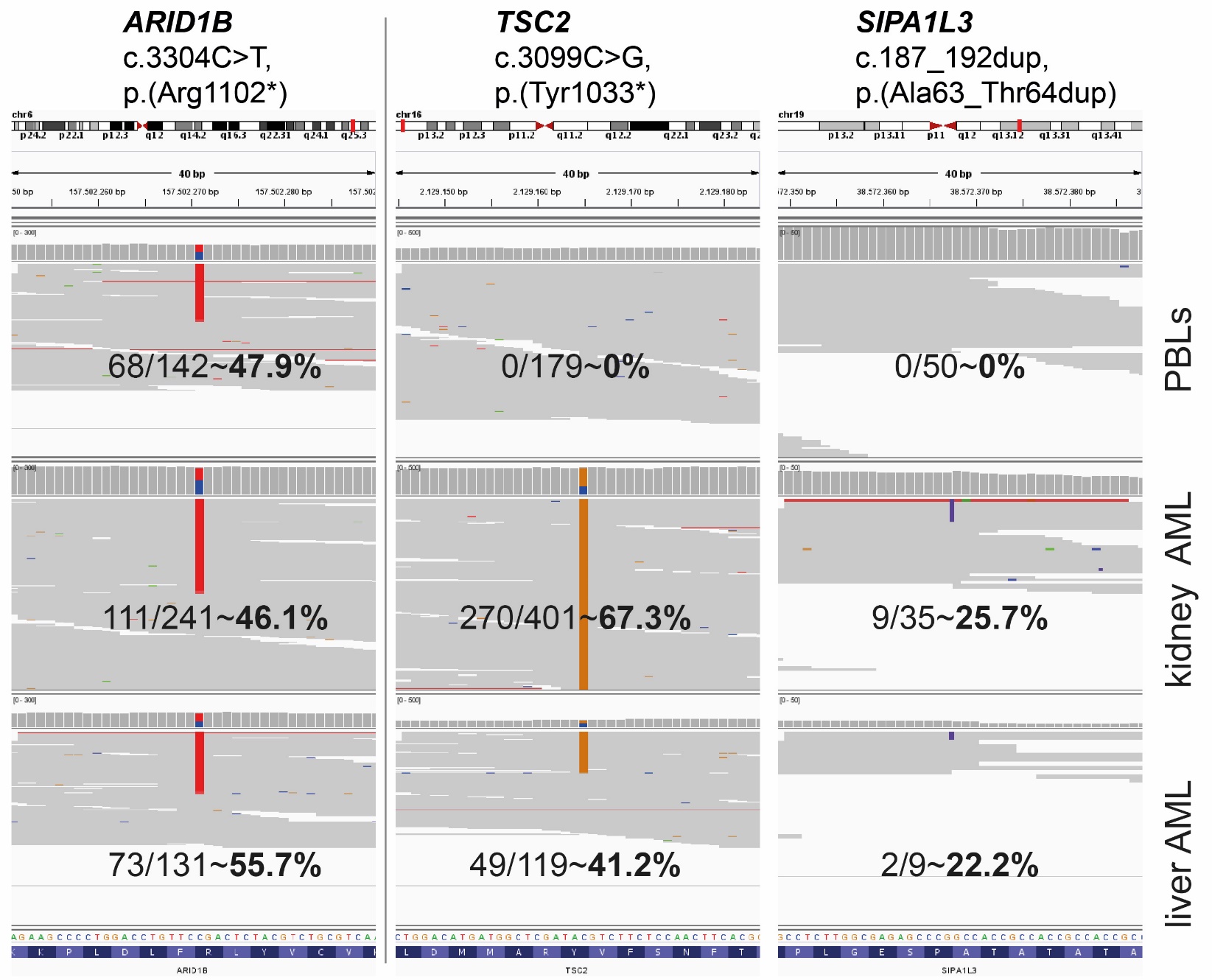
Fig. S1 | IGV snapshots from exome sequencing**

Left panel shows the previously known germline variant in *ARID1B*: chr6[hg19]:g.157502271C>T (NM_020732.3: c.3304C>T, p.(Arg1102*)). Both kidney and liver AML show no significant deviation from the expected allele fraction (AF) of 50% (Fisher's exact test: p ~ 0.75 for kidney AML vs PBLs, p ~ 0.23 for liver AML vs PBLs).

Right panel shows the post zygotic variants in *TSC2* and *SIPA1L3,* identified in both AMLs. Assuming complete tumor pureness (or complete monoclonality) the kidney AML shows a significant deviation from the expected AF of 50 % for the *TSC2* variant c.3099C>G, p.(Tyr1033*) (NM_000548.3; chr16[hg19]:g.2129165C>G) (two-sided binominal test: p < 0.001) while the liver AML does not significantly deviate from this expectation (two-sided binominal test: p ~ 0.07). This indicates a loss of heterozygosity at the *TSC2* locus in the epithelioid kidney AML. The *SIPA1L3* gene locus is GC-rich and repetitive and thus covered relatively low in all exomes. Also, the variant c.187_192dup, p.(Ala63_Thr64dup) (NM_015073.2) is a relatively complex indel showing evidence for reference bias in mapping. Given theses factors we did not calculate deviance from expected AF for this variant. However, it was identified and confirmed by Sanger sequencing in both tumors and absent from the PBL sample.

**
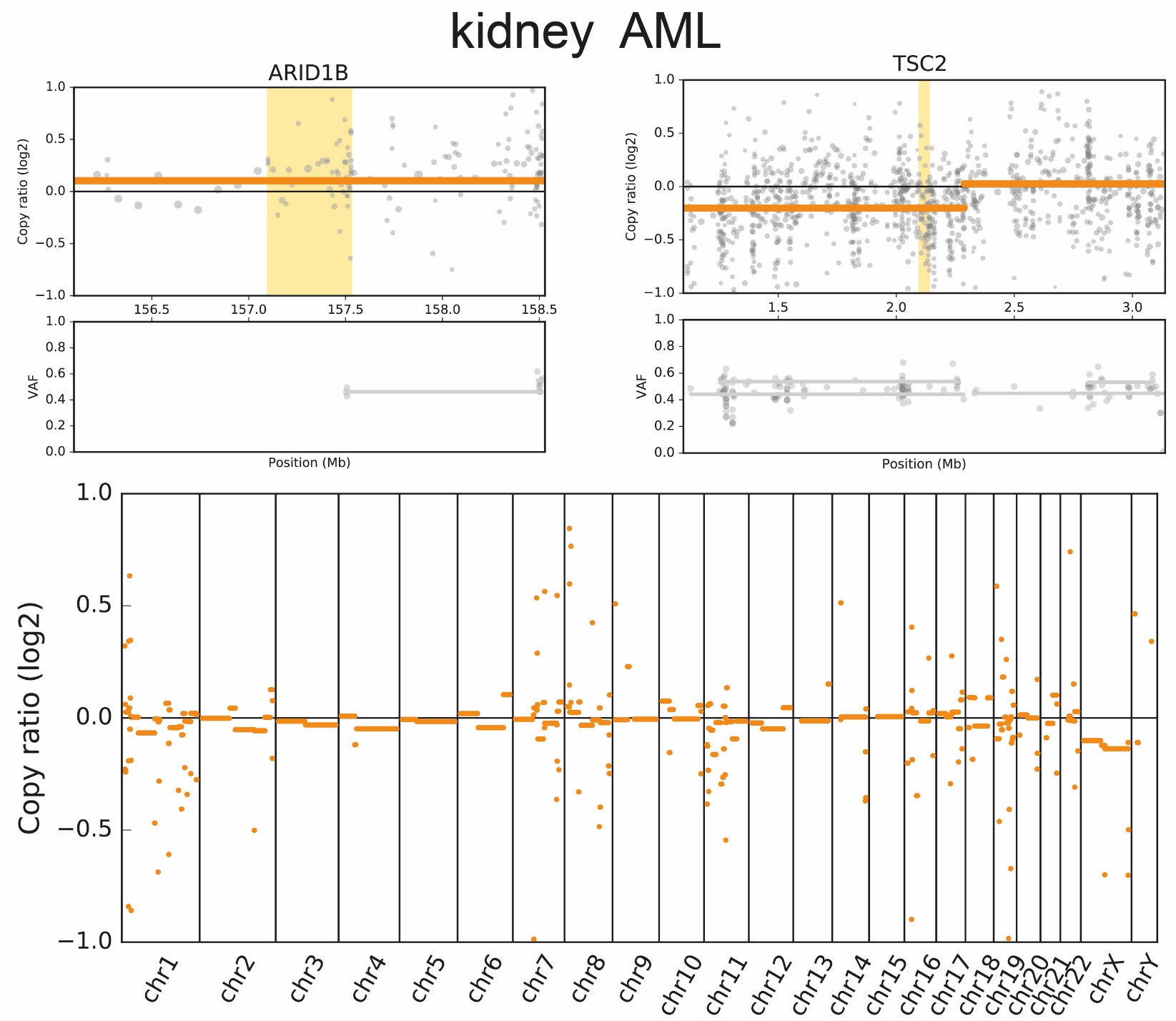
Fig. S2 | Copy number analysis from exome data from the kidney AML using CNVkit**

At the *ARID1B* locus no evidence for a CNV or LOH was found. At the *TSC2* locus the profile indicated a deletion in a subpopulation of tumor cells. However, this is not supported by allele distribution for the heterozygous variant (VAF). LOH would be indicated by a VAF above 0.7 or below 0.3, while it is around 0.5 at the *TSC2* locus (see also Additional file 2). The genome wide copy number profile showed no large significant alterations. Also, no differences between the profile of the kidney AML and the liver AML sample was evident (compare Fig. S5). Orange and grey dots represent markers used by the CNVkit algorithm (target and anti-target regions). Shading of grey dots indicates weight within the analysis for the respective marker. Orange bars represent copy number segmentation calls, and grey bars represent segmentation calls for the variant allele frequencies used for LOH analysis.

**
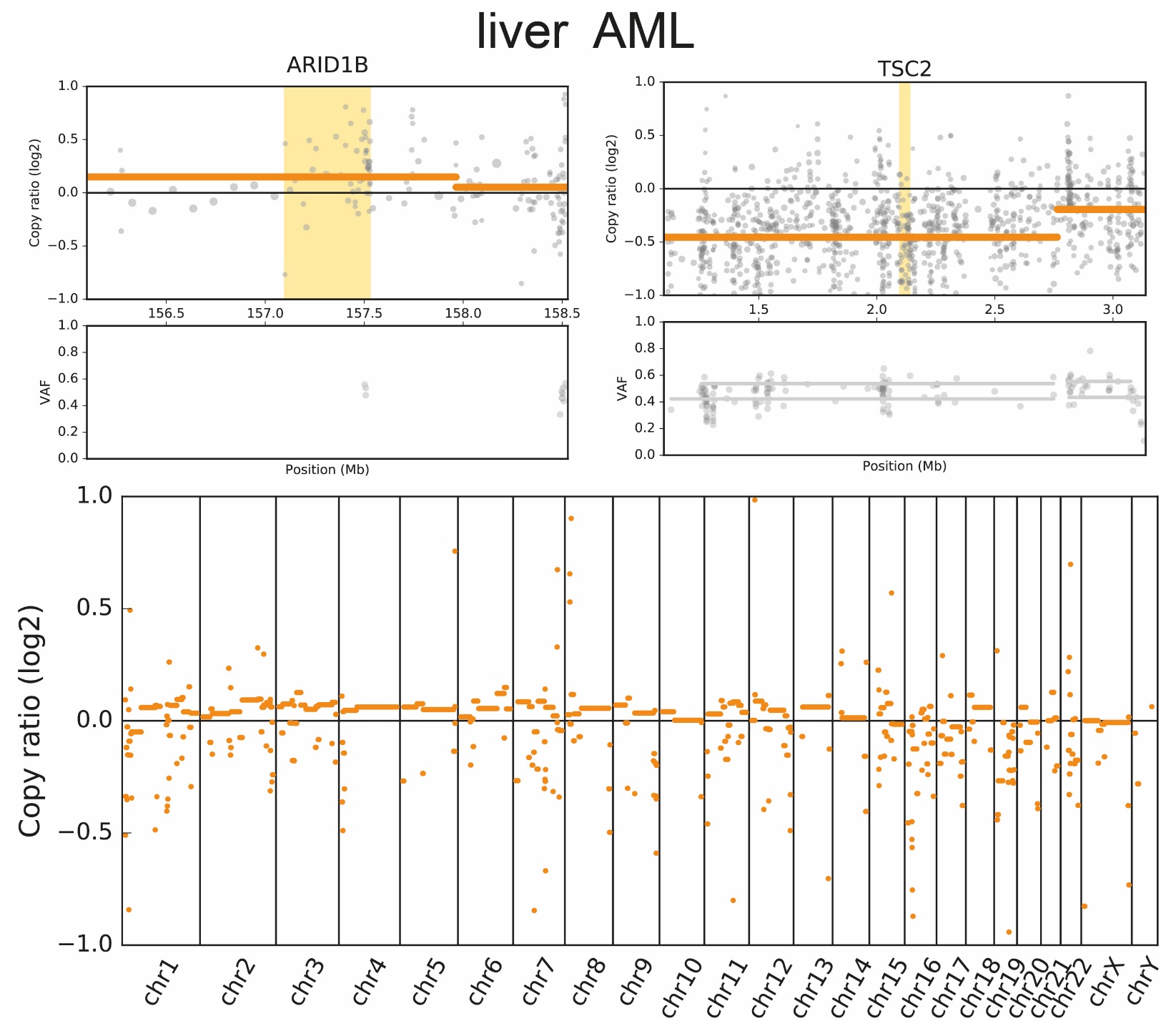
**

**Fig. S3 | Copy number analysis from exome data from the liver AML using CNVkit**

At the *ARID1B* locus no evidence for a CNV or LOH was found. At the *TSC2* locus the profile indicated a deletion in a subpopulation of tumor cells. However, this is not supported by allele distribution for the heterozygous variant (VAF). LOH would be indicated by a VAF above 0.7 or below 0.3, while it is around 0.5 at the *TSC2* locus (see also Additional file 2). The genome wide copy number profile showed no large significant alterations. Also, no differences between the profile of the liver AML here and the kidney AML sample was evident (compare Fig. S4). Orange and grey dots represent markers used by the CNVkit algorithm (target and anti-target regions). Shading of grey dots indicates weight within the analysis for the respective marker. Orange bars represent copy number segmentation calls, and grey bars represent segmentation calls for the variant allele frequencies used for LOH analysis.


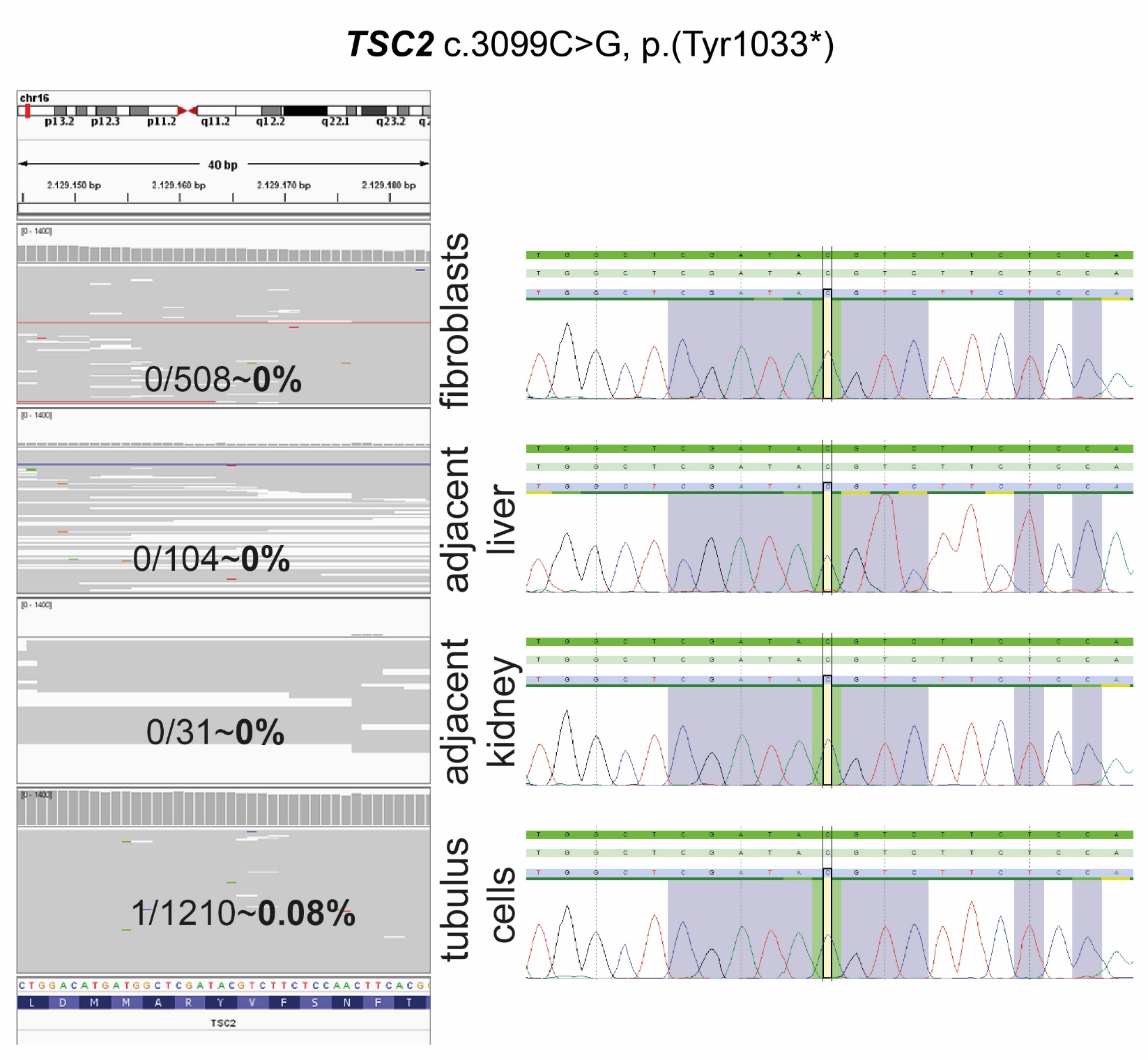


**Fig. S4 | IGV snapshots from targeted Cancer Panel and Sanger sequencing**

Left panel shows IGV snapshots for the variant c.3099C>G, p.(Tyr1033*) (NM_000548.3; chr16[hg19]:g.2129165C>G) from TruSight Cancer Panel sequencing for the four unaffected tissues studied. Right panel shows confirmatory results from Sanger sequencing as displayed in SeqPilot software (version 4.1.2). In the tubulus cells one read of 1210 showed evidence for the alternative G-allele. However, this was considered an artefact read (see Fig. S3).

**
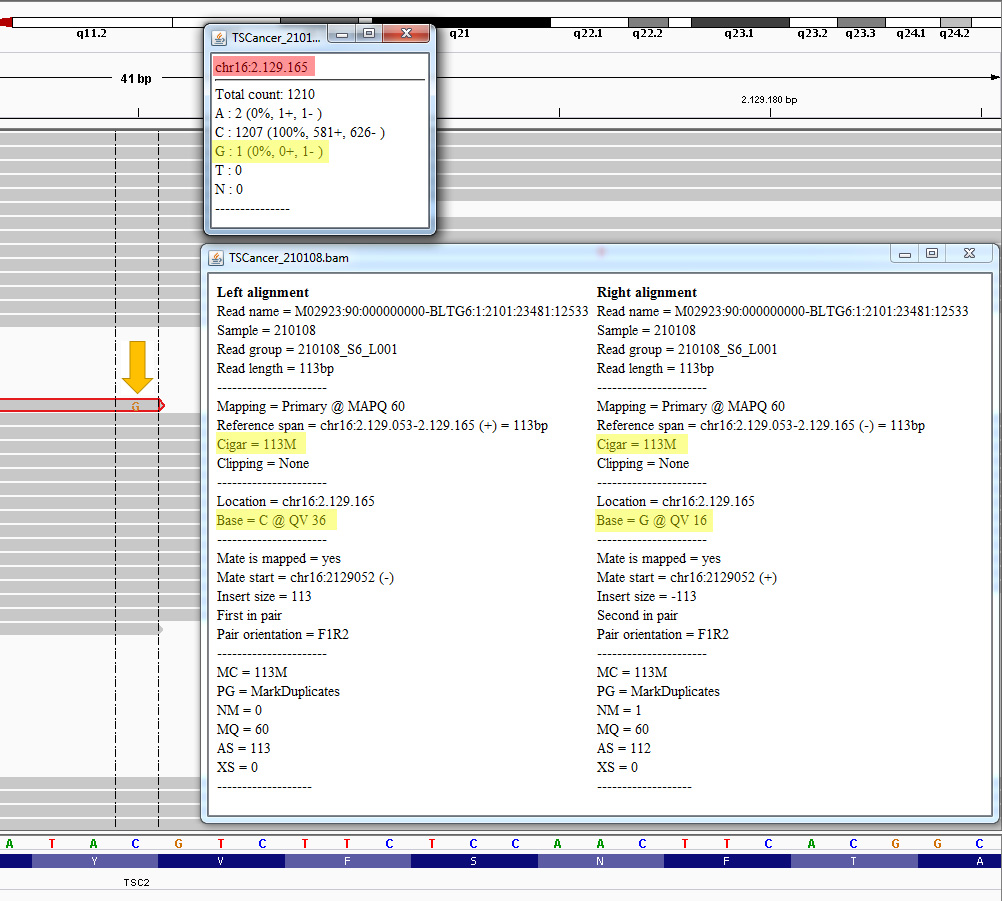
Fig. S5 | IGV snapshot of the artefact read identified in tubulus cells**

Due to bad base quality (PHRED scaled QV=16; yellow arrow and highlights) and because it was only supported by one of the reads in the overlapping read pair (right alignment) this read was considered an artefact. The overlapping second read in the pair showed a wildtype C-allele at the position (QV=36). Also note that the read pair is only 113 base pairs (bp) long although the panel was sequenced as 150 bp paired-end on an Illumina MISeq. This indicates that the read pair originated from a short DNA fragment which sequenced into the adapters and was therefore clipped.
